# Inhibition of MAD2L1 Mediates Pulmonary Fibrosis through Impairment of Mitochondrial Function and Induction of Cell Senescence

**DOI:** 10.1155/2022/9663354

**Published:** 2022-10-04

**Authors:** Lan Wang, Ruyan Wan, Xinyu Chen, Xiaoshu Guo, Zhongzheng Li, Weiming Zhao, Peishuo Yan, Guoying Yu

**Affiliations:** State Key Laboratory Cell Differentiation and Regulation, Henan International Joint Laboratory of Pulmonary Fibrosis, Henan Center for Outstanding Overseas Scientists of Pulmonary Fibrosis, College of Life Science, Henan Normal University, Xinxiang, Henan, China

## Abstract

Idiopathic pulmonary fibrosis (IPF) is a chronic, irreversible, and progressive interstitial lung disease characterized by recurrent alveolar epithelial cell injury, fibroblast hyperproliferation, and cumulative deposition of extracellular matrix leading to alveolar destruction in the lungs. Mitotic arrest deficient 2 like 1 (MAD2L1) is a component of the mitotic spindle assembly checkpoint that prevents the onset of anaphase until all chromosomes are properly aligned at metaphase and is a potential therapeutic target in cancers. However, the role of MAD2L1 in pulmonary fibrosis has not been explored. We analyzed the expression of MAD2L1 in lung tissues from control subjects, IPF patients, and mice with bleomycin-induced fibrosis via IHC, qRT-PCR, and Western blot analysis. We examined the roles of MAD2L1 in ROS production, mitochondrial function, cell senescence, and the establishment of a profibrotic microenvironment. We found that MAD2L1 was highly upregulated in alveolar epithelial cells in fibrotic lung tissues from both patients with IPF and mice with bleomycin-induced fibrosis. Loss of MAD2L1 expression or activity led to decreases of cell viability and proliferation in A549 cells. Subsequent mechanistic investigation demonstrated that inhibition of MAD2L1 damaged mitochondria, which led to augmented ROS production and cellular senescence, and thus promoted the establishment of a profibrotic microenvironment. Taken together, these results reveal that alleviation of alveolar epithelial cell mitochondrial damage arising from augmentation of MAD2L1 may be a novel therapeutic strategy for mitigating pulmonary fibrosis.

## 1. Introduction

Idiopathic pulmonary fibrosis (IPF) is a chronic, irreversible, and progressive interstitial lung disease characterized by recurrent alveolar epithelial cell injury, fibroblast hyperproliferation, and cumulative deposition of extracellular matrix leading to alveolar destruction in the lungs [1–3]. Emerging evidence suggests that mitochondrial dysfunction plays important roles in the initiation and progression of multiple aged-related lung diseases, including IPF [4]. Mitochondrial biogenesis, ROS production, mitochondrial DNA (mtDNA) leakage, mitosis, and mitophagy [5] have been identified in lung epithelial cells, fibroblasts, and macrophages; these changes result in maladaptation to cellular stress, increasing susceptibility to activation of profibrotic responses [6–9].

The mitotic cycle of normal cells is strictly regulated by multiple checkpoints. If those checkpoints are broken, the loss or incorrect distribution of chromosomes during uncontrolled mitosis will result in the formation of aneuploid cells [10]. The spindle assembly checkpoint (SAC) ensures the fidelity of chromosome inheritance by ensuring that sister chromosomes form bipolar attachments with microtubules of the mitotic spindle [11]. MAD2L1 has two conformations: closed MAD2L1 (C-Mad2) and open MAD2L1 (O-Mad2) [12, 13]. Mad2 binds directly to Cdc20 during mitosis and inhibits the anaphase-promoting complex or cyclostome (APC/C), thus inhibiting the process of cell division [14–16]. MAD2L1 induces aneuploidy of cell chromosomes and then promotes tumor formation [17]. MAD2 depletion triggers premature cellular senescence in human primary fibroblasts [18]. Currently, there is limited information available on the role of MAD2L1 in fibrotic diseases.

The abnormal expression of MAD2L1 in our fibrotic lung meta-dataset drove us to explore the role of MAD2L1 in the progression of pulmonary fibrosis. In this study, we aimed to uncover the role of MAD2L1 in pulmonary fibrosis to find a novel therapeutic target for IPF treatment.

## 2. Materials and Methods

### 2.1. Mouse Lungs with Bleomycin-Induced Fibrosis and Human IPF Lung Tissues

Eight to 10-week-old female (C57BL/6) mice were obtained from Weitonglihua Experimental Animal Technology Co. Ltd. On day 0, the mice were anesthetized with 40% isoflurane diluted in 1,2-propanediol; and then intratracheally instilled with bleomycin at a dose of 1.5 U/kg diluted in PBS or with PBS only (sham). The animals were sacrificed with 100 mg ketamine/kg body weight and 10 mg xylazine/kg body weight intraperitoneally, and samples were collected on day 14 for further analysis after bleomycin treatment. The animal maintenance and handling procedures followed the Henan Normal University Institutional Animal Care and Use Committee (IACUC, SMKX-2118BS1018) guidelines, which coordinate with the Association of Animal Behavior and National Regulations.

The IPF lung tissues and normal controls were recruited based on the ATS/ERS/JRS/ALAT Clinical Practice Guidelines at Henan Provincial Chest Hospital. The study was approved by the Henan Provincial Chest Hospital Medical Research Ethics Committee (No. 2019-05-07), and informed consent was obtained from all the patients before surgery.

### 2.2. Cell Culture and Treatment

The A549 cell line was obtained from the American Type Culture Collection (Shanghai, China) and cultured in a DME/F-12 medium supplemented with 10% (v/v) heat-inactivated FBS, 1% penicillin, and streptomycin in a 37°C humidified incubator with 5% CO_2_. When the cells reached approximately 80% confluence, the cells were digested with 0.25% trypsin/EDTA solution. The digested cells were plated into 6-well plates. A549 cells were transfected with small interfering RNA by using Lipofectamine 3000 when the cells reached approximately 70% confluence according to the manufacturer's instructions. In addition, the cells were treated with M21I-1(100 *μ*M), an inhibitor of MAD2L1.

### 2.3. Quantitative Real-Time PCR (qRT-PCR)

For gene transcription analysis of mouse lungs and A549 cells, the total mRNA was extracted using RNeasy mini kits according to manufacturer's instructions. Reverse transcription of the RNA was performed using Prime Script reverse transcriptase. qRT-PCR was carried out using a Light Cycler 96 fluorescent quantitative PCR system (Roche) and SYBR Premix Ex Taq with the cDNA as the template. The conditions for the qRT-PCR were as follows: initial denaturation at 95°C for 10 min followed by 40 cycles of 95°C for 15 s, 60°C for 1 min, and 72°C for 30 s. The average Ct values from the triplicate analyses were normalized by the average Ct values of GAPDH. The ratios of the expression levels were calculated with the 2−ΔΔCt method. The primer pairs for the genes in this study are described in Table 1.

### 2.4. Western Blot Analysis

Total proteins were extracted from mouse lung tissues or cells using RIPA buffer (Beyotime) containing protease inhibitors (Beyotime). The protein concentration was evaluated with the Pierce BCA method. Then, each protein sample was separated by 8–12% SDS-PAGE, and the proteins were transferred to nitrocellulose membranes. After blocking in 5% skim milk, the membranes were probed with the following primary antibodies overnight at 4°C: anti-MAD2L1 (1 : 3,000, # A300-301A, Bethyl), anti-*β*-actin (Affinity), anti-Pink1 (1 : 2000, #T0022, Affinity), anti-LC3 (ab192890, # ab192890, Abcam), anti-MFN1 (1 : 1000, #14739, Cell Signaling Technology), anti-MFN2 (1 : 1000, #ab124733, Abcam), anti-Pink1(1 : 1000, #AF7581, Affinity), anti-OPA1 (1 : 1000, #DF8587, Affinity), anti-p53 (1 : 1000, #2527, Cell Signaling Technology), anti-phospho-p53 (1 : 1000, # AF3037, Affinity), anti-p21 (1 : 1000, #2947, Cell Signaling Technology), anti-phospho-p21 (1 : 1000, #AF3290, Affinity), anti-collagen1 (1 : 1000, # ab34710, Abcam), anti-E-cadherin (1 : 1000, # ab2786, Abcam), anti-N-cadherin (1 : 1000, #13116, Cell Signaling Technology), and anti-Vimentin (1 : 1000, # BF8006, Affinity). The membranes were then incubated with a horseradish peroxidase-conjugated secondary antibody for 1 h at room temperature. The blots were visualized by using an enhanced chemiluminescence system (Bio-Rad, USA).

### 2.5. Immunohistochemistry

Briefly, mouse lung tissues were embedded in paraffin, sectioned, and rehydrated. Then, the antigens were recovered by microwave heating of histologic sections in citrate buffer at 95°C for 10 min. The endogenous peroxidase was neutralized using endogenous peroxidase blocking solution. Afterward, the lung sections were incubated with a primary antibody at 4°C overnight. This step was followed by incubation with biotin-labeled secondary antibodies at 37°C for 30 min. Next, the lung sections were developed with DAB working solution and then counterstained with hematoxylin and mounted with mounting medium. Images were obtained with a microscope (Nikon, Japan).

### 2.6. Cell Counting Kit-8 Assay

Briefly, cells were seeded at a density of 5000 cells/well in 96-well microplates. Following incubation, the cells were treated with siRNAs or M21I-1 (100 *μ*M) for 24 h or 48 h. After treatment, 10 *μ*L of CCK-8 reagent was added to each well and gently mixed. Finally, the optical density was measured at 450 nm by a microplate reader (Thermo Fisher Scientific). Prism7 software was used to analyze the data.

### 2.7. Wound-Healing Assay

A549 cells were seeded into a sterile 6-well plate and cultured overnight in an incubator. The cells were transfected with siRNA or treated with M21I-1 (100 *μ*M). When the cells had grown to reach 90–100% confluence in a cell monolayer, a straight line was scratched on the cell surface with a sterile pipette tip. The wounds were photographed at 0 h, 12 h, and 24 h postwounding under an inverted research microscope (Leica D-35578, Wetzlar, Germany).

### 2.8. EdU Assay

Cell proliferation was measured with an EdU assay kit (Ribobio). Cells were seeded into 24-well plates at 2 × 10^4^ cells per well. After 24 h of the treatment, each well was incubated with 300 *μ*l of 50 *μ*M EdU medium for 2 h at 37°C. Then, the cells were fixed in 4% paraformaldehyde for 30 min and permeabilized with 0.5% Triton X-100 for 10 min. After washing with PBS, the cells were incubated with 1 × Apollo reaction cocktail for 30 min. Subsequently, 1 × Hoechst 33342 was added, and the cells were incubated for additional 30 min. The positive cells were assessed under fluorescence microscopy (Nikon, Japan).

### 2.9. ROS and Mitochondrial Membrane Potential (MMP)

For determination of the ROS and MMP levels, A549 cells were treated with siRNAs or M21I-1 for 24 h, and then the ROS and MMP levels in the A549 cells were determined with detection kits according to the manufacturer's instructions. The cells were observed under a fluorescence microscope, and the results were analyzed using Image J software.

### 2.10. Senescence-associated *β*-galactosidase (SA-*β*-Gal)

The activity of SA-*β*-gal was determined using a *β*-galactosidase staining kit (Beyotime) following the manufacturer's instructions. Briefly, the treated cells were rinsed with PBS and then fixed in a fixative solution at room temperature for 10 min. After being washed, the cells were incubated with the staining solution at 37°C overnight. The positive cells were observed under a microscope, and the results are expressed as the percentage of total cells.

### 2.11. Statistical Analysis

The data are shown in bar graphs showing the mean ± standard deviation. The significance of the differences between the two groups was analyzed using two-tailed Student's *t*-tests. All statistical analyses were performed using GraphPad Prism7. ^*∗*^*P* < 0.05, ^*∗∗*^*P* < 0.01, ^*∗∗∗*^*P* < 0.001, and ^*∗∗∗∗*^*P* < 0.0001 were considered to indicate statistical significance.

## 3. Results

### 3.1. MAD2L1 Expression was Upregulated in IPF Lungs and Bleomycin-Induced Fibrotic Mouse Lungs

**Figure 1:**
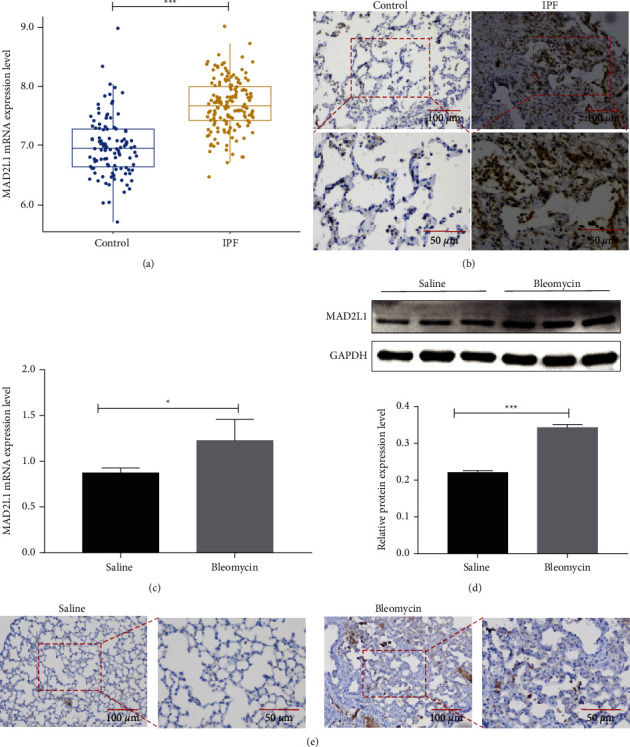
MAD2L1 was upregulated in IPF lungs and the bleomycin-induced mouse lungs. (a) mRNA level of MAD2L1 in lung tissues from patients with IPF and healthy subjects. (b) Representative images of MAD2L1 immunohistochemical staining in sections of lung tissue from patients with IPF and healthy subjects. The boxed regions are shown at higher magnification in the lower panels. (c) mRNA expression of MAD2L1 in bleomycin- and saline-treated mice as determined by qRT-PCR analysis. (d) Protein expression of MAD2L1 in bleomycin- and saline-treated mice as determined by WB analysis. The data are shown as the mean ± SEM, *n* ≥ 3 per group. ^*∗*^*P* < 0.05; ^*∗∗∗*^*P* < 0.001; ^*∗∗∗∗*^*P* < 0.0001. (e) Representative images of MAD2L1 immunohistochemical staining in sections of lung tissue from bleomycin-treated mice and saline-treated control mice. The boxed regions are shown at higher magnification in the lower panels.

To determine the role of MAD2L1 in pulmonary fibrosis, the level of MAD2L1 in IPF and a bleomycin model of lung fibrosis in mice were examined. *MAD2L1* mRNA (Figure 1(a)) level was significantly higher in the lungs of patients with IPF than those in healthy subjects (data from GSE47460). MAD2L1 was mainly expressed in the alveolar epithelium surrounding the fibrotic area and macrophages in lungs from patients with IPF and was weakly expressed in normal alveolar epithelium (Figure 1(b)), it also demonstrated that *MAD2L1* was a dramatically upregulated in type II alveolar epithelial cells in IPF Cell Atlas Data (https://www.IPFcellatlas.com). In line with the human data, the expression of *Mad2l1* in mRNA (Figure 1(c)) and protein (Figure 1(d)) levels were induced in bleomycin-induced fibrotic mouse lungs, Mad2l1 was primarily expressed in the alveolar epithelium, and macrophages too in the lungs of fibrotic mice (Figure 1(e)).

### 3.2. Proliferation of A549 Cells was Inhibited by the MAD2L1 siRNA or Inhibitor

**Figure 2:**
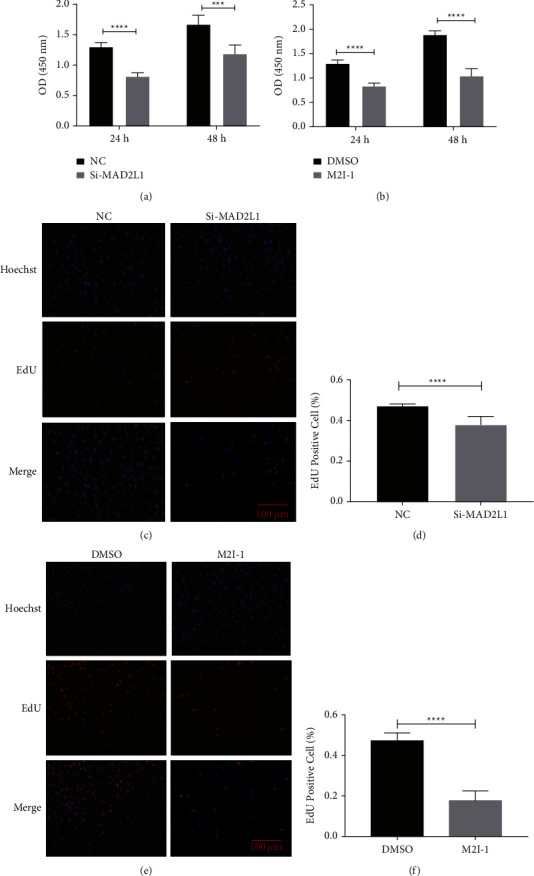
MAD2L1 is critical for A549 cell proliferation. (a) A549 cell viability in a time course of treatment with MAD2L1 siRNA or control siRNA. (b) A549 cell viability in a time course of treatment with M2I-1 or the vehicle control. (c, d) Effect of MAD2L1 siRNA or control siRNA on A549 cell proliferation at 24 hours. (e, f) Effect of treatment with M2I-1 on A549 cell proliferation at 24 and 48 hours. ^*∗∗∗*^*P* < 0.001; ^*∗∗∗∗*^*P* < 0.0001.

To investigate the role of MAD2L1 in cellular viability *in vitro*, we transfected A549 cells with a MAD2L1-targeting siRNA and administrated of M21I-1 (100 *μ*M), an inhibitor of MAD2L1. We found that inhibition of the MAD2L1 expression in A549 cells led to a strong reduction in viability, as measured by CCK-8 assay (Figure 2(a)). Adding the inhibitor of MAD2L1 M21I-1 had similar results (Figure 2(b)). In addition, we further determined if the decreased MAD2L1 altered the cellular proliferation of A549 cells. As shown in Figures 2(c) and 2(d), MAD2L1 suppression led to significantly decreased proliferative activity in A549 cells, as measured by EdU assay. Moreover, M21I-1 decreased proliferative activity in A549 cells (Figures 2(e) and 2(f)). These data collectively suggest that MAD2L1 affects the proliferation of A549 cells.

### 3.3. Migration of A549 Cells Was Inhibited by the MAD2L1 siRNA or Inhibitor

**Figure 3:**
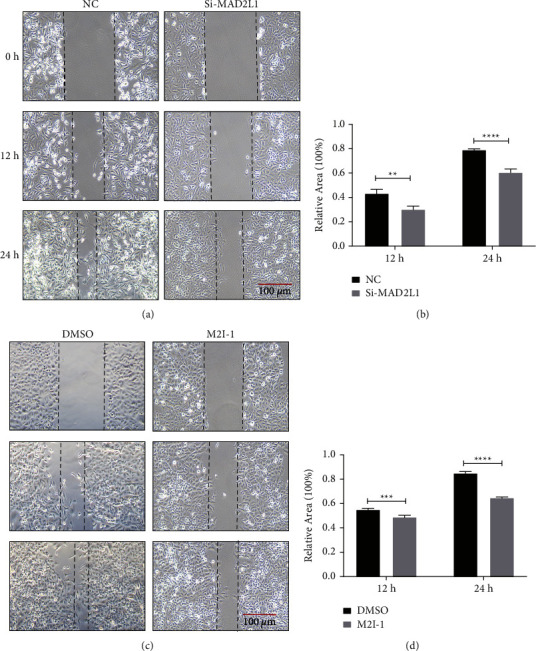
Migration of A549 cells is inhibited by MAD2L1 siRNA or a MAD2L1 inhibitor. (a) A scratch assay was performed on A549 cells following transfection with MAD2L1 siRNA or control siRNA. (b) Quantification of A549 cell wound healing following transfection with MAD2L1 siRNA or control siRNA. (c) A scratch assay was performed on A549 cells following M2I-1 or control treatment. (d) Quantification of A549 cell wound healing in the presence or absence of the inhibitor. ^*∗∗*^*P* < 0.01; ^*∗∗∗*^*P* < 0.001; ^*∗∗∗∗*^*P* < 0.0001.

We examined the effect of MAD2L1 inhibition on cell migration and found that the repress MAD2L1 by siRNA significantly impaired wound healing of A549 cells (Figure 3(a)). The scratch reparability was calculated by Image J software (Figure 3(b)). In addition, a similar result was observed in the presence of M2I-1 compared to DMSO (the control) (Figures 3(c) and 3(d)). These results indicate that MAD2L1 inhibition negatively affects the migration of A549 cells.

### 3.4. Inhibition of MAD2L1 Impaired Mitochondrial Structure and Function

**Figure 4:**
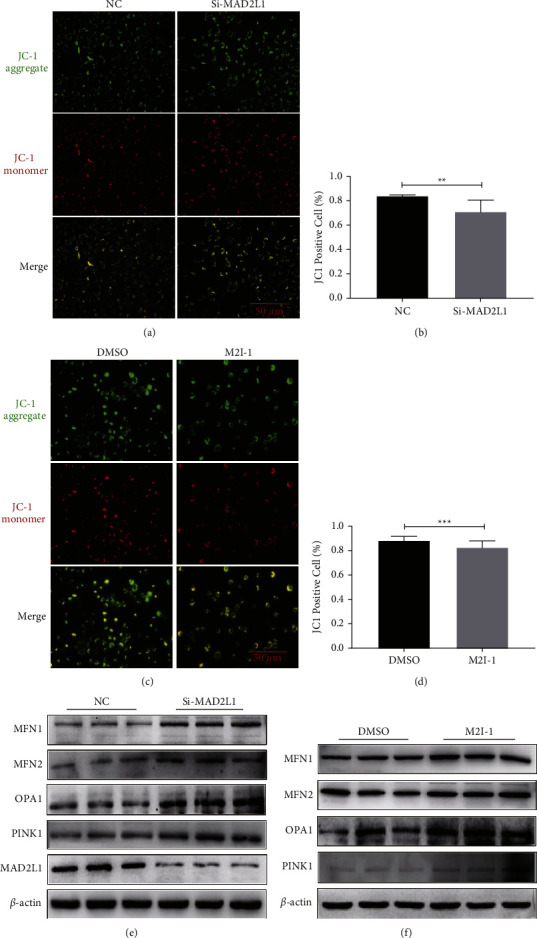
MAD2L1 inhibition impaired mitochondrial structure and function. (a) The MMP levels of A549 cells transfected with MAD2L1 siRNA or control siRNA were measured by JC-1 staining. (c) The MMP levels of A549 cells were detected by JC-1 staining following treatment with M2I-1 or DMSO (control). (b, d) Quantitative analysis of the MMP percentage. The MMP percentage was calculated as the ratio of JC-1 aggregates to monomers. (e) The expression of MFN1, MFN2, OPA1, and PINK1 was determined by western blot analysis. *β*-actin was used as the loading control. (f) Western blot analysis was performed to confirm the alterations in MFN1, MFN2, OPA1, and PINK1 in total protein extracts after treatment with M2I-1 or DMSO (control).

To test the effects of MAD2L1 on mitochondrial structure and function in A549 cells, the MMP in A549 cells was determined after the cells were transfected with MAD2L1 siRNA or treated with M2I-1. The results showed that a significantly loss of MMP in response to MAD2L1 suppression (Figures 4(a)–4(d)), and this decrease was accompanied by increased MFN1/2 and OPA1 as well as PINK1 in A549 cells (Figures 4(e) and 4(f)). These results suggest that MAD2L1 was essential for maintaining the membrane potential of mitochondria and stabilizing mitochondrial structure and function.

### 3.5. Inhibition of MAD2L1 Increased ROS Production in A549 Cells

**Figure 5:**
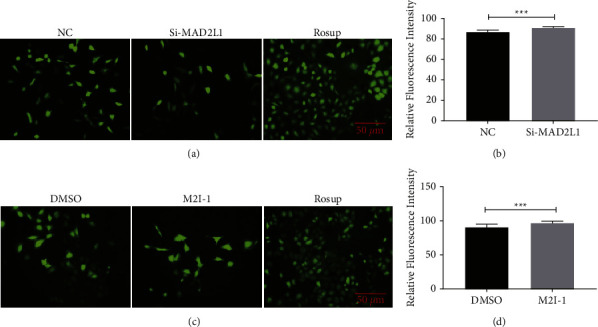
MAD2L1 inhibition increased ROS production in A549 cells. (a) ROS was measured using DCFH-DA surface fluorescence in A549 cells transfected with MAD2L1 siRNA or control siRNA. (b) Quantification of intensity of the DCFH-DA surface fluorescence. (c) ROS was measured using DCFH-DA surface fluorescence in A549 cells treated with M2I-1 or DMSO (control). (d) Quantification of intensity of the DCFH-DA surface fluorescence. ^*∗∗∗*^*P* < 0.001; ^*∗∗∗∗*^*P* < 0.0001.

To investigate the effect of MAD2L1 on ROS generation, we detect the ROS production in A549 cells after inhibition MAD2L1. As shown in Figures 5(a) and 5(b), the fluorescence intensity was obviously more intensive in the transfected/treated cells than in the control cells. According to the absolute quantification values, treatment/transfection significantly enhanced the release of ROS compared with that in the group (Figures 5(c) and 5(d)).

### 3.6. Alteration of MAD2L1 Expression Led to A549 Cell Senescence

**Figure 6:**
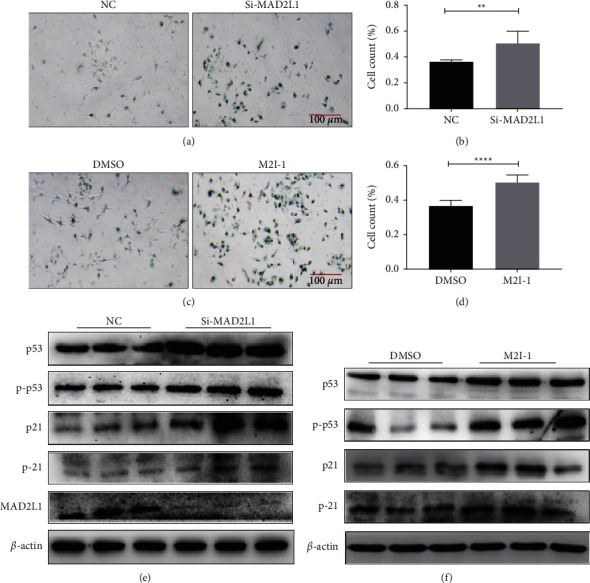
Inhibition of MAD2L1 augmented the senescence of A549 cell. (a) Cell senescence was detected by SA-*β*-Gal staining following transfection with MAD2L1 siRNA or control siRNA. (b) The data are presented as the % of *β*-gal-positive cells/total cells. (c) SA-*β*-Gal staining was applied to detect cellular senescence following treatment with the MAD2L1 inhibitor M2I-1 or control. (d) The data are presented as the % of *β*-gal-positive cells/total cells. (e) The expression of MAD2L1 and aging-related marker proteins (serine-20-phosphorylated p53, p53, threonine-145-phosphorylated p21, and p21) were determined by western blot analysis. *β*-actin was used as the control. (f) Western blot analysis was performed to confirm the alterations in serine-20-phosphorylated p53, p53, threonine-145-phosphorylated p21, and p21. *β*-actin was used as the control. ^*∗*^*P* < 0.05; ^*∗∗∗∗*^*P* < 0.0001.

To further delineate the role of MAD2L1 in ATII cell senescence, we examined the SA-*β*-gal activity and age-related gene expression after transfection of A549 cells with MAD2L1 siRNA or nontargeting siRNA. The results showed that silencing MAD2L1 with siRNA significantly increased SA-*β*-gal activity (Figures 6(a) and 6(b)), and the expression of p53, phos-p53, p21, and phos-p21 was upregulated (Figure 6(e)). This observation was consistent with inhibition of MAD2L1 with the inhibitor M2I-1 (Figures 6(c), 6(d), and 6(f)). These data confirm that inhibition of MAD2L1 induces p53-p21 pathway activation and promotes ATII cell senescence.

### 3.7. MAD2L1 Inhibition Increased the Synthesis of Extracellular Matrix Proteins

**Figure 7:**
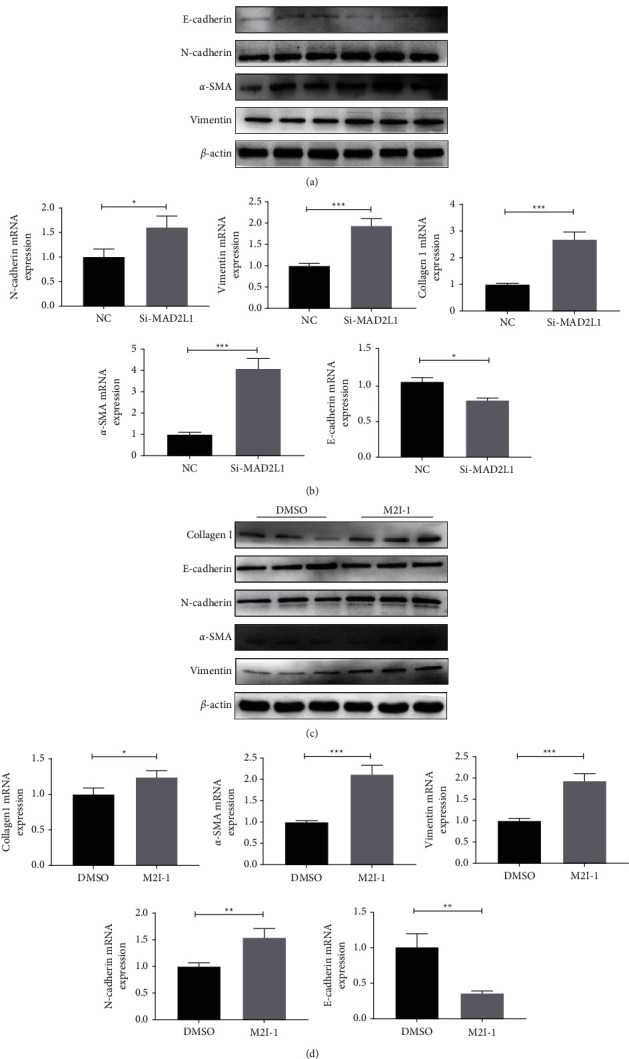
MAD2L1 inhibition increased the synthesis of extracellular matrix proteins. (a) The expression of E-cadherin and the ECM-related proteins Collagen1, N-cadherin, *α*-SMA, and Vimentin was detected by western blot analysis in A549 cells transfected with MAD2L1 siRNA or control siRNA. (b) The expression of E-cadherin and the ECM-related genes Collagen1, N-cadherin, *α*-SMA, and Vimentin were determined by qRT-PCR in A549 cells transfected with MAD2L1 siRNA or control siRNA. (c) qRT-PCR analysis of Collagen1, E-cadherin, N-cadherin, *α*-SMA, and Vimentin mRNA expression in A549 cells treated with M2I-1 or DMSO (control). (d) Western blot analysis was performed to confirm the alterations in Collagen1, E-cadherin, N-cadherin, *α*-SMA, and Vimentin in total protein extracts from cells treated with M2I-1 or DMSO (control).

Next, we determined whether decreased MAD2L1 could alter the ECM production in A549 cells. As shown in Figure 7(a), inhibition of MAD2L1 led to marked increases in Collagen1 and *α*-SMA levels and upregulation of N-cadherin and Vimentin, which are biomarkers for mesenchymal cells. In addition, inhibition of MAD2L1 caused loss of epithelium marker E-cadherin. Consistent with these results, the mRNA levels of *Collagen1*, *α-SMA*, *N-cadherin,* and *Vimentin* were increased in A549 cells (Figure 7(b)). In addition, M2I-1 treatment resulted in marked increases in ECM and EMT-related proteins and genes (Figures 7(c) and 7(d)). Taken together, these results demonstrate that the profibrogenic effects of MAD2L1 are mediated through increased synthesis of extracellular matrix proteins.

## 4. Discussion

The incidence of IPF is increasing worldwide, yet the pathogenesis needs further exploration [19]. IPF is characterized by deposition of extracellular matrix proteins that modify normal lung physiology [20, 21]. Alveolar epithelial cells play important roles in defense and immunomodulatory functions.

In this study, we demonstrated that MAD2L1 plays a crucial role in the pathogenesis of pulmonary fibrosis. The baseline MAD2L1 was low in normal lung tissues but was increased in fibrotic lungs of both human subjects with IPF and mice challenged with bleomycin. IHC staining of lung sections revealed increased MAD2L1-positive cells in both IPF patients and bleomycin-treated mice. We first demonstrated that depleting MAD2L1 in A549 cells inhibited viability and proliferation, indicating that the repair ability of alveolar epithelial cells was reduced. However, ineffective alveolar epithelial cell repair can lead to aberrant fibroblastic responses and induce fibrosis [22]. Therefore, we hypothesized that MAD2L1 inhibition may augment lung fibrosis.

Under normal conditions, mitochondria are dynamic organelles that constantly undergo fusion and fission events to regulate metabolism, cell proliferation, and survival [23]. Abnormality of mitochondrial fusion can cause mitochondrial fragmentation and hyperfusion [24]. Mitochondria initiate a protective mechanism to control and maintain their quality in mild stress, with the participation of Mfn1/2 and OPA1, the fused mitochondria undergo rapid material exchange and restore their own functions, overcoming their dysfunction through diffusion and sharing of components between the organelles [25]. In contrast, severe or prolonged stress leads to increased ROS production, inflammatory responses, and cellular senescence [23]. In this study, we found that MAD2L1 inhibition decreased the MMP in A549 cells compared with that in the control cells. MAD2L1 inhibition can augment mitochondrial dysfunction and thus contribute to the pathogenesis of pulmonary fibrosis. In addition, MAD2L1 siRNA or M2I-1 treatment led to marked upregulation of Mfn1, Mfn2, and OPA1. Studies have suggested that mitochondrial autophagy (mitophagy) plays an important role in eliminating damaged mitochondria [25, 26]. Next, we found that PINK1 was upregulated in A549 cells with MAD2L1 inhibition, MAD2L1 depletion *in vitro* caused upregulation of PINK1 which may be viewed as a failed attempt to prevent fibrosis progression.

ROS are thought to play a dual role in many physiological and pathophysiological processes. At low concentrations, ROS exhibit beneficial effects by regulating cellular proliferation, differentiation, intracellular signaling, and homeostasis. In contrast is the high level of ROS damage intracellular proteins, lipids, and nucleic acids [27, 28]. Recent studies have implicated ROS in the pathogeneses of aging and lung diseases, some of which include IPF, COVID-19, chronic obstructive lung disease, and lung cancer [29–31]. ROS can activate TGF-*β* signaling either directly or indirectly via the activation of proteases [32]. We found that knockdown of MAD2L1 in A549 cells led to accelerated production of ROS compared to that in the control cells. This may promote EMT and induce pulmonary fibrosis. Furthermore, generation of ROS in alveolar epithelial cells mediate mitochondrial DNA damage, which increased the susceptibility to lung fibrosis [30]. The mitochondrial dysfunction, in turn, leads to the augmentation of ROS production, inflammatory responses, and cellular senescence and thus induces pulmonary fibrosis [23].

As mitochondrial dysfunction is a feature of cellular senescence, and as IPF is an age-related disease, we further examined the relationship between MAD2L1 and cellular senescence. Mitochondrial ROS drive senescence [33], scavenging mtROS with the mitochondria-targeted antioxidant MitoQ, or acetyl-l-carnitine delays cellular senescence [23]. We further tested the effects of MAD2L1 inhibition on ATII cell senescence, silencing MAD2L1 and M2I-1-treated significantly increased SA-*β*-gal activity as well as protein levels of p53 and p21. We conclude that inhibition of MAD2L1 will lead to mitochondrial dysfunction and thus to cellular senescence. One of the driving forces behind fibrosis is a process in which epithelial cells lose epithelial proteins including E-cadherin and gain mesenchymal markers such as N-cadherin and Vimentin [34]. Both the mRNA and protein levels of ECM proteins and N-cadherin and Vimentin increased after inhibition of MAD2L1. In addition, MAD2L1 downregulation led to loss of E-cadherin. Altogether, MAD2L1 inhibition promotes the establishment of a profibrotic microenvironment, thus inducing pulmonary fibrosis.

## 5. Conclusions

Overall, the present study revealed that MAD2L1 was upregulated in both IPF lungs and the lungs of mice with bleomycin-induced fibrosis. Loss of MAD2L1 expression and activity led to decreased cell viability and proliferation in A549 cells. Inhibition of MAD2L1 led to mitochondria damage, augmentation of ROS production, and cellular senescence, and induction of extracellular matrix thus promoted pulmonary fibrosis. The data suggested that alleviation of mitochondrial damage in alveolar epithelial cells through augmentation of MAD2L1 may be served as a novel therapeutic strategy for a cure of idiopathic pulmonary fibrosis.

## Figures and Tables

**Table 1:** Genes selected for expression analysis.

Primer name	Oligonucleotide sequence (5′-3′)
M-GAPDH-F	5′-GTCAGGTCATCACTATCGGCAAT-3′
M-GAPDH-R	5′-AGAGGTCTTTACGGATGTCAACGT-3′
M-MAD2L1-F	5′-GTGGCCGTTTTTCTCATTTG-3′
M-MAD2L1-R	5′-AGGTGAGTCTATCTGCACT-3′
H-GAPDH-F	5′-CCACCCATGGCAAATTCC-3′
H-GAPDH-R	5′-GATGGGATTTCCATTGATGACA-3′
H-MAD2L1-F	5′-GGACTCACCTTGCTTGTAACTAC-3′
H-MAD2L1-R	5′-GATCACTGAACGGATTTCATCCT-3′

## Data Availability

The data used to support the findings of this study are included within the article.

## Abbreviations

IPF: Idiopathic pulmonary fibrosis
MAD2L1: Mitotic arrest deficient 2 like 1
ROS: Reactive oxygen species
qRT-PCR: Quantitative real-time PCR
WB: Western blot
IHC: Immunohistochemistry
TGF-*β*1: Transforming growth factor *β*1
mtDNA: Mitochondrial DNA
SAC: Spindle assembly checkpoint
MAD1L1: Mitotic arrest deficient 1-like 1
BUBR1: Benzimidazole related 1
BUB1: Benzimidazole 1
MPS1: Multipolar spindle 1
APC/C: Anaphase-promoting complex or cyclostome
CCK8: Cell counting kit-8 assay
MMP: Mitochondrial membrane potential
SA-*β*-gal: Senescence-associated *β*-galactosidase
ATII: Type II alveolar epithelial cells
MFN: Mitofusin
OPA1: Optic atrophy 1
PINK1: Mitophagy--associated protein putative kinase 1.
